# Influence of PVP and PEG on the Electrochemical Synthesis of Magnesium Hydroxide

**DOI:** 10.3390/ma18122917

**Published:** 2025-06-19

**Authors:** Shengqing Wang, Fangyang Liu, Zongliang Zhang, Jun Wang, Liangxing Jiang

**Affiliations:** 1 School of Metallurgy and Environment, Central South University, Changsha 410083, China; wangshengqing@gem.com.cn (S.W.); liufangyang@csu.edu.cn (F.L.); zongliang.zhang@csu.edu.cn (Z.Z.); 2 Centre Research Institute, GEM Co., Ltd., Shenzhen 518000, China; 3 School of Chemistry and Chemical Engineering, Changsha University of Science and Technology, Changsha 410114, China

**Keywords:** Mg(OH)_2_, electrodeposition, PVP, PEG, magnesium chloride

## Abstract

The functional performance of magnesium hydroxide (Mg(OH)_2_) is intrinsically governed by its crystallographic morphology. Herein, we demonstrate an electrochemical deposition strategy to synthesize Mg(OH)_2_ from abandoned MgCl_2_ resources in salt lakes, achieving simultaneous waste valorization and morphology control. Systematic investigations were conducted on the effects of polyvinylpyrrolidone (PVP) and polyethylene glycol (PEG) as surfactants on electrochemical parameters (cell voltage, pH, current efficiency, and energy consumption) and morphological evolution (XRD, SEM, and laser particle size analysis). Results show that the cell voltage and pH increased proportionally with surfactant concentration, with a current efficiency of 93.86% and an optimal energy consumption of 4.15 kW h·t^−1^ at an optimal PVP concentration of 6 g·L^−1^. PEG addition exhibited a similar trend in process parameter modulation. Morphological evolution analysis revealed that appropriate PEG dosage promoted the transformation of irregular Mg(OH)_2_ flakes into near-spherical platelets, accompanied by a measurable increase in particle size. This work establishes structure–property relationships between surfactant molecular design and Mg(OH)_2_ crystallization, providing theoretical support for the controllable electrochemical preparation of magnesium hydroxide with different morphologies. Furthermore, it opens up a novel and innovative technical pathway to promote the high-value utilization of abandoned magnesium resources in salt lakes.

## 1. Introduction

Magnesium hydroxide (Mg(OH)_2_) is an environmentally friendly inorganic functional material with promising applications in various fields due to its unique layered crystal structure and exceptional physicochemical properties [1,2]. With a low corrosivity (pH ≈ 10.4) [3], high thermal stability (decomposition temperature > 300 °C) [4], and wide bandgap (5.4 eV) [5,6], Mg(OH)_2_ is well-suited for industrial wastewater treatment [7,8], polymer flame retardancy [9], and biomedical applications [10]. Its significance is further underscored in the context of carbon neutrality [11]. For example, in industrial wastewater treatment, Mg(OH)_2_ can neutralize up to 3.7 mmol g^−1^ [12]. In polymer flame retardancy, incorporating 40% Mg(OH)_2_ allows materials to meet the UL94 V-0 flame-retardant standard [13]. In biomedicine, Liu et al. demonstrated that surface-modified Mg(OH)_2_ nanoparticles exhibit an inhibition rate of over 95% against Escherichia coli and Staphylococcus aureus, with negligible cellular toxicity, suggesting their potential as antibacterial dressings or drug carriers [14,15].

Mg (OH)_2_ has demonstrated outstanding performance in pollutant adsorption in applications, such as wastewater treatment and exhaust gas purification [11,12,16]. Chanda et al. documented that nano-flake Mg (OH)_2_ exhibits a remarkable adsorption capacity for Pb^2+^, reaching 620 mg g^−1^ with an adsorption efficiency exceeding 95% [17]. The two-dimensional nanosheet structure of Mg (OH)_2_ offers a substantial specific surface area of up to 525 m^2^ g^−1^ [18]. This elevated specific surface area, in conjunction with enhanced surface activity, results in exceptional adsorption capabilities for heavy metal ions (e.g., Pb^2+^, Cd^2+^) and organic pollutants (e.g., dyes, phenols). Additionally, the hexagonal lamellar morphology significantly enhances interfacial compatibility with polymers and boosts the flexural strength of composites by more than 40% [10,12].

The preparation technology of Mg(OH)_2_ typically involves the precipitation–hydrothermal method. Nanotubes (30–50 nm in diameter) and hexagonal sheets (<20 nm in thickness) have been successfully prepared by incorporating surfactants like polyvinylpyrrolidone (PVP) and polyethylene glycol (PEG) [12,19,20]. For example, Zheng et al. [21] utilized PEG-8000 as a morphology-controlling agent to synthesize hexagonal Mg(OH)_2_ sheets ranging from 20 to 50 nm in thickness, effectively inhibiting the growth rate of the (001) crystal surface with a growth rate ratio of 1.67. Fang’s group [22] synthesized hexagonal nanosized Mg(OH)_2_ particles with an average size of 174 nm through the adsorption effect of PVP, achieving a low standard deviation of 12% in particle size distribution and mitigating particle agglomeration. Furthermore, Deng et al. [23] fabricated nanosheet Mg(OH)_2_ with a specific surface area of up to 525 m^2^·g^−1^ using graphene as a substrate, exhibiting an adsorption capacity for methyl orange (MO) dye of 450 mg·g^−1^, indicating excellent pollutant adsorption performance. However, the conventional hydrothermal method presents significant drawbacks, including harsh reaction conditions (180 °C, 12 h) high energy consumption, and equipment costs comprising over 60% of production expenses [24]. These factors significantly limit its industrial feasibility. Recent studies have shown that the cost of upscaling the current laboratory preparation process increases by 8–10 times, posing a challenge in meeting the increasing demand for the comprehensive utilization of magnesium resources from salt lakes [25,26].

This study proposes an innovative electrochemical morphology control strategy using salt lake brine to achieve directional deposition of Mg^2+^ through a diaphragm electrolysis system. This method relocates the surfactant action field to the electrode interface from the conventional hydrothermal kettle. It capitalizes on the selective adsorption effect of PVP and PEG on the cathode surface, where the difference in adsorption energy can reach up to 0.38 eV. By modulating the crystal growth rate ratio of (001) and (101), this approach achieves the adjustability of morphology. Pan et al. demonstrated the electrolysis of MgCl_2_ aqueous solutions for Mg(OH)_2_ preparation, achieving a maximum current efficiency of 88.93% and unit energy consumption of 10.05 kWh·kg^−1^ under optimized conditions (150 g·L^−1^ MgCl_2_, 125 mA·cm^−2^ current density, 45 °C, and 180 min electrolysis) [27]. Yang et al. employed a hydrothermal method with 3 wt% PEG-8000 as a morphology control agent to synthesize hexagonal Mg(OH)_2_ platelets with regular morphology and excellent dispersion [20]. Surface-grafted modifications further endowed these platelets with hydrophobicity and compatibility with polymeric matrices. Fang et al. utilized a double-injection hydrothermal approach, achieving nanoscale Mg(OH)_2_ with regular hexagonal morphology and superior dispersibility at a PVP loading of 4.0 wt% and 3.0 mol·L^−1^ MgCl_2_ concentration, highlighting the flame-retardant performance of the product [22]. In comparison to the traditional process, this technology offers three significant advantages: (1) reduction in the reaction temperature to 60 °C, leading to a decrease in energy consumption [28], (2) regulation of product morphology by the surfactant of PVP and PEG [29], and (3) utilization of bischofite, a by-product from lithium extraction in salt lakes, for the preparation of Mg(OH)_2_ with a MgCl_2_ content exceeding 96.00% [26]. By introducing a novel electrochemical shape control approach, this research addresses the constraints of conventional hydrothermal techniques, enhancing the efficiency of preparing Mg(OH)_2_ and optimizing resource utilization. This innovative method demonstrates potential for not only offering a new method for the effective production of Mg(OH)_2_ but also paving the way for the high-value utilization of magnesium resources found in salt lakes.

## 2. Materials and Methods

### 2.1. Materials

Bischofite (MgCl_2_·6H_2_O, Qinghai Western Magnesium Co., Ltd., Delingha, China) was obtained from Qarhan Salt Lake Magnesium Salt Mine in Qinghai. The primary raw material for this experiment was salt lake bischofite, with its main components and contents detailed in Table 1. Prior to the electrolysis experiment, the raw material underwent pretreatment. Specifically, MgCl_2_·6H_2_O was dissolved in laboratory-prepared deionized water to yield a Mg^2+^ solution with a concentration of 100 g·L^−1^. The solution was then filtered three times to remove insoluble matter before being utilized in the electrolysis experiment. PVP and PEG-4000 (McLean Biochemistry Science and Technology Co., Ltd., (Shanghai, China) were used as surfactants during the electrodeposition experiment. Laboratory-grade pure water was used for sample dissolution and electrodeposition experiments.

### 2.2. Experimental Method

**Figure 1:**
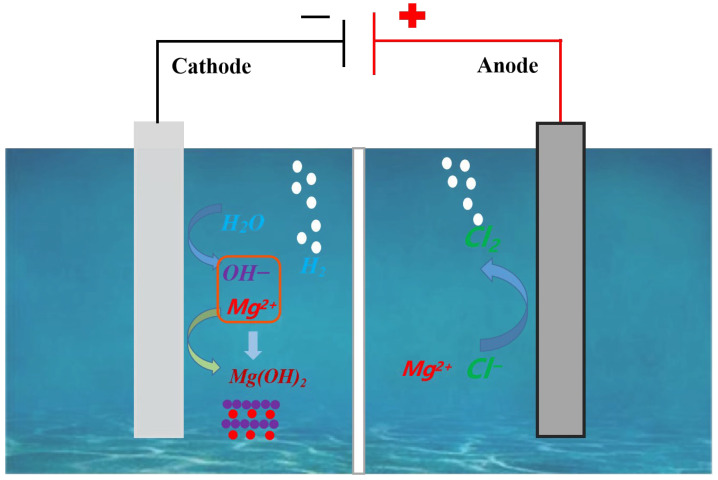
Presents a schematic illustration of the apparatus utilized for the electrochemical synthesis of Mg(OH)_2_.

Figure 1 presents a schematic illustration of the apparatus utilized for the electrochemical synthesis of Mg(OH)_2_. The electrodeposition preparation of Mg(OH)_2_ was carried out using an Electrolytic Cell (acrylic material, Huazhong Organic Glass Products Factory, Wuhan, China) with an electrolyte capacity of 500 mL and an acid- and alkali-resistant diaphragm (505G-150GY, Liaoning Bolian Filtration Co., Ltd., Tieling, China) in the center. Specific information on the diaphragm is shown in Table 2; the anode plate was made of titanium coated with a ruthenium–iridium catalyst (Ti–ruthenium–yttrium oxide, Suzhou Shutai Industrial Technology Co., Ltd., Suzhou, China), while the cathode was made of a stainless steel plate (304 stainless steel, Institute of High Purity Metal Materials, Hefei, China). The MgCl_2_ solution was added to the cathode and anode chambers of the electrolyzer, and the current density was set to 100 mA cm^−2^ after connecting to the constant current power supply. The initial concentration of MgCl_2_ in the electrolyte was 100 g L^−1^, and the electrode plate spacing was 3.0 cm. The electrolysis temperature was maintained at 40 °C in a water bath, and the electrolysis reaction continued for 4 hours. At the end of electrolysis, the precipitated slurry in the cathode chamber was removed, filtered, washed with deionized water, and dried. Finally, the Mg(OH)_2_ was characterized for its physical and chemical properties.

The current density dictates the quantity of electrons transferred during electrolysis, directly influencing the deposition rate of Mg(OH)_2_ at the cathode. A low current density results in slow precipitation, whereas an excessively high current density induces rapid Mg(OH)_2_ growth near the cathode—both scenarios impeding morphological control of the product. As demonstrated by Pan et al., current efficiency peaks and specific energy consumption reaches a minimum within the 100–150 mA cm^−2^ range, with energy consumption increasing at higher densities. Integrating critical experimental variables (solution concentration, temperature, electrode material, and pH), the current density was set at 100 mA/cm^2^ in this study to optimize the balance between morphology control and electrochemical efficiency. The principle of preparing Mg(OH)_2_ by electrodeposition using magnesium chloride from salt lakes can be represented by the following electrode reaction.

Anode: 2Cl^−^ → Cl_2_↑ + 2e^−^ (1)

Cathode: 2H_2_O + 2e^−^ → 2OH^−^ + H_2_↑ (2)

Anodic chamber: Mg^2+^ + 2OH^−^ → Mg(OH)_2_↓ (3)

Total reaction: MgCl_2_ + 2H_2_O → Mg(OH)_2_↓ + Cl_2_↑ + 2H_2_↑ (4)

### 2.3. Analytical Characterization

The analytical equipment used in this electrodeposition experiment included a SevenCompact precision pH meter (S210, Mettler Toledo Technology (China) Co., LTD., Shanghai, China), used for pH measurements, a powder X-ray diffractometer (XRD, using Cu Kα radiation, PANalytical Empyren, Spectris Instrumentation and Systems Shanghai Ltd., Shanghai, China), used for physical phase analysis of samples, a scanning electron microscope (SEM, TESCAN S9000, Tescan China Ltd., Shanghai, China), used for morphological characterization of samples, and a laser particle size analyzer (SCF-108A, OMEC, Zhuhai Oumeike Instrument Co., LTD., Zhuhai, China), used for the particle size distribution tests. Current efficiency and unit energy consumption were calculated according to Formulas (5) and (6) as follows:

(5) $$\eta =\frac{NF*n_{Mg(OH)2}}{I*t}\times 100\%$$

(6) $$W=\frac{\int_{0}^{t}UIdt}{m_{{mg(OH)}_{2}}}$$

where **n**_Mg(OH)2_ is the mol amount of Mg(OH)_2_ prepared by electrodeposition, *F* is the Faraday constant (96,485 C mol^−1^), *N* is the number of electrons transferred per mol of product, *I* is the current intensity set for the electrolysis process (A), *U* is the cell voltage (V), and *t* is the electrolysis time.

## 3. Results and Discussion

### 3.1. Effect of Surfactant Addition on Electrolytic Parameters

**Figure 2:**
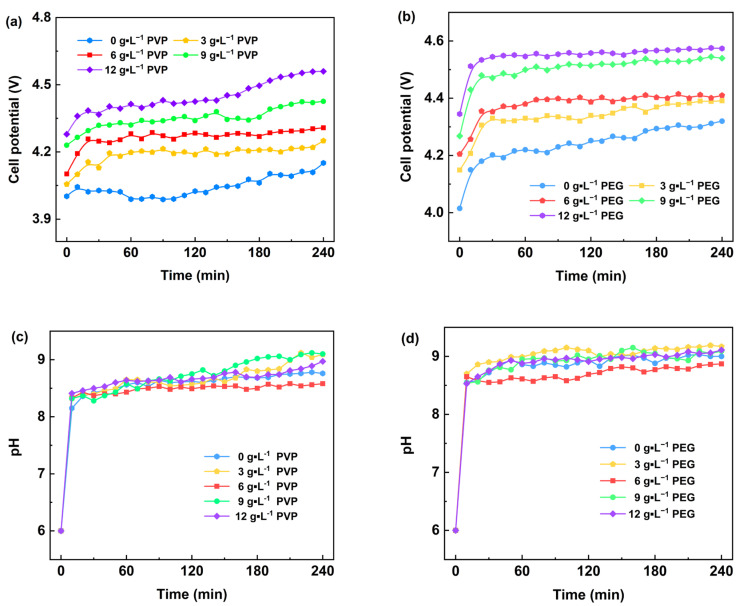
Effect of different surfactants on cell voltage (**a**,**b**) and pH value (**c**,**d**) in the electrodeposition process.

Figure 2 illustrates the variations in cell voltage and pH in the cathode chamber during electrolysis under different surfactant concentrations in the electrolyte. Figure 2a shows that with the increase in PVP content and the prolongation of the electrolysis time, the cell voltage increased continuously, and its average value rose from 4.04 V to 4.44 V. This may be attributed to the addition of PVP, which changed the ionic transport properties of the electrolyte and increased the resistance of ionic transport, leading to an increase in the cell voltage. Meanwhile, Figure 2c shows that with the increase in PVP content, the pH value of the cathode chamber solution changes minimally; however, with the prolongation of the electrolysis time, the overall pH value of the cathode chamber solution exhibits an increasing trend and ultimately stabilizes between 8.5 and 9.0. This indicates that the addition of PVP has a small effect on the acidity and alkalinity of the cathode chamber solution, while the electrolysis process itself leads to a gradual alkalization of the cathode chamber solution, which ultimately reaches a dynamic equilibrium.

**Figure 3:**
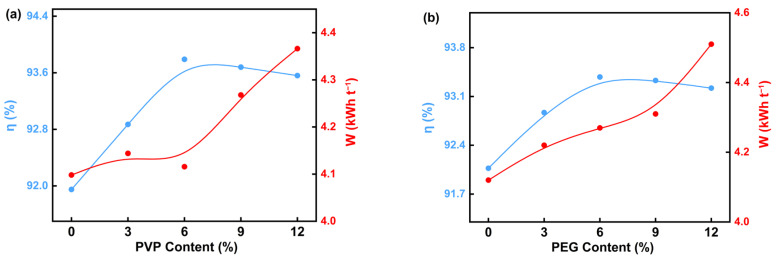
Effect of different PVP (**a**) and PEG (**b**) on current efficiency and energy consumption in the electrodeposition process.

Figure 3a shows that the current efficiency first increases and then gradually decreases with the increasing content of PVP in the electrolyte. The current efficiency reaches the maximum value of 93.80% at the addition of 6 g·L^−1^ of PVP, while the unit energy consumption increases with increasing PVP content, with the optimum unit energy consumption at this point being 4.15 kWh t^−1^. This result indicates that the appropriate amount of PVP can effectively improve the current efficiency of the electrolysis process; however, excessive PVP will lead to a decrease in the current efficiency and, at the same time, increase the unit energy consumption. This may be due to the fact that an appropriate amount of PVP enhances the interfacial properties of the electrolyte, promoting the transport and deposition of ions, thus increasing current efficiency, while an excessive amount of PVP may increase the viscosity of the electrolyte, hindering ion transport, which consequently decreases current efficiency and increases energy consumption.

Figure 2b demonstrates the trend of cell voltage changes when PEG is added to the electrolyte. With the increase in PEG content, the cell voltage of the electrolysis system rises continuously. During the initial phase of the electrolysis experiment (within the first 30 min), the cell voltage increases rapidly and then remains stable. This suggests that the addition of PEG significantly changes the electrochemical behavior of the electrolyte, especially at the early stage of electrolysis, and PEG may lead to a rapid increase in the cell voltage by affecting the ion transport path or the interfacial properties. In contrast, the effect of PVP on the cell voltage was characterized by a gradual increase with increasing content and prolonged electrolysis time, exhibiting relatively smooth changes. This may be attributed to the dispersion and stability of PEG in the electrolyte, which has a relatively small hindering effect on ion transport; however, with the increase in content, this hindering effect is gradually apparent. Figure 2d shows that the addition of PEG has a minimal effect on the pH of the cathode chamber because PEG affects the electrochemical process mainly by changing the physical properties of the electrolyte rather than directly participating in the electrode reaction.

Figure 3b indicates the effect of PEG content on current efficiency and unit energy consumption. With the increase in PEG content, the current efficiency initially rises and then gradually declines, reaching a maximum value of 93.38% at the PEG addition of 6 g·L^−1^. This result indicates that moderate amounts of PEG can significantly improve electrolysis efficiency, while an excessive amount of PEG reduces current efficiency. This may be due to the fact that the concentration of PEG in the electrolyte is too high, which impedes ion transport, thereby decreasing current efficiency. Meanwhile, unit energy consumption rises with the increase in PEG content, indicating that the appropriate amount of PEG content plays an important role in enhancing electrolysis efficiency and minimizing energy consumption.

As shown in Figure 2 and Figure 3, the cell voltage of the electrolysis process increases with the increase in PVP and PEG content. This is mainly because the increase in the content of PVP and PEG as surfactants significantly increases the viscosity of the electrolyte. The increase in viscosity leads to a decrease in ion mobility in the solution, which makes the electrolysis process require a higher voltage to drive ion migration, thus leading to an increase in cell voltage. Furthermore, with the extended electrolysis time, Mg(OH)_2_ was deposited near the electrode plates in the cathode chamber, which further increased the impedance of the solution. Simultaneously, the total number of conductive ions in the electrolyte decreased due to the deposition effect, which also led to an increase in cell voltage to some extent. During electrolysis, the rising pH of the cathode chamber was due to the dynamic equilibrium established between the precipitation of Mg(OH)_2_ and the production and consumption of OH^−^. With the prolongation of electrolysis time, the pH of the cathode chamber solution gradually reached a steady state. This suggests that the OH^−^ concentration in the solution can be maintained at a relatively stable level during the electrolysis process despite the generation of Mg(OH)_2_ precipitation, resulting in a stabilized pH.

The trends in current efficiency and unit energy consumption during electrolysis were consistent. As the concentrations of PVP and PEG increased, the current efficiency initially increased and then gradually decreased, while unit energy consumption increased with higher surfactant content. This is due to the fact that the conductivity and ion migration efficiency of the electrolyte are optimized when surfactants are added in moderate amounts, which improves the current efficiency. However, at excessively high surfactant concentrations, the increased viscosity of the electrolyte impedes ion migration and reduces the conductivity of the electrolyte. Simultaneously, the increase in solution impedance makes the electrolysis process require a higher cell voltage to maintain the electrolysis reaction, which results in an increase in unit energy consumption. The addition of PVP and PEG to the electrolyte significantly influenced cell voltage, cathode chamber pH, current efficiency, and unit energy consumption during the electrolysis process. These effects mainly originated from the modification of ion migration behavior by surfactants and the impact of Mg(OH)_2_ precipitation on solution conductivity and impedance during electrolysis.

### 3.2. Effect of PVP on the Properties of Electrodeposition Product

**Figure 4:**
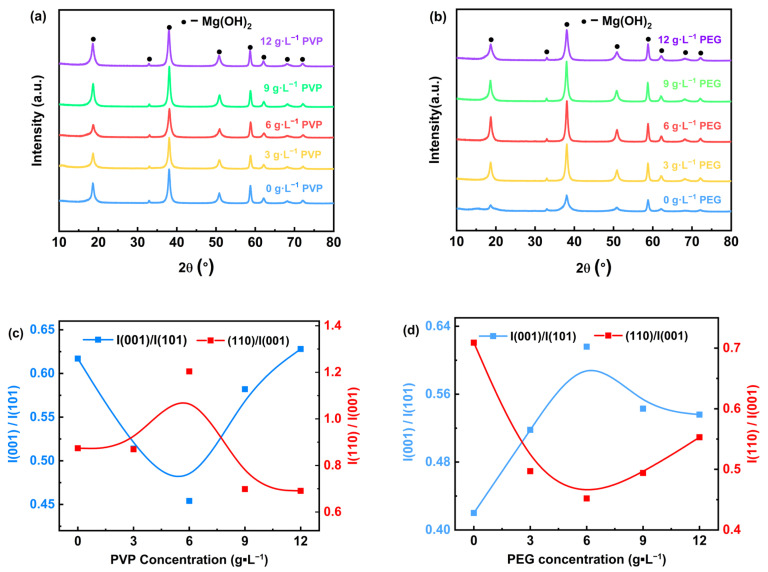
XRD patterns (**a**,**b**) and peak intensity ratios (**c**,**d**) of Mg(OH)_2_ with different surfactants.

The results of the physical phase analysis of Mg(OH)_2_ prepared by the electrodeposition method using MgCl·6H_2_O sourced from salt lakes are presented in Figure 4. The X-ray diffraction results of the electrodeposited samples with different concentrations of PVP added to the electrolyte solution are presented in Figure 4a, showing that the diffraction peaks of Mg(OH)_2_ with typical hexagonal structure were observed, which correspond to the diffraction crystal planes noted in the standard reference JCPDS Card No. 07-0239 (001), (100), (101), (102), (110), (111), (103), and (200) planes, respectively. Notably, no diffraction peaks of other phases were detected. The 2θ values of the strongest diffraction peaks (h k l) are located at 18.52°, 37.98°, and 58.66°, corresponding to the diffracted crystal planes (001), (101), and (110). The results indicate that the prepared Mg(OH)_2_ have excellent crystallinity. Figure 4c shows the X-ray peak intensity ratios of (001) nonpolar and (101) polar facets, as well as the (110) polar and (001) nonpolar facets in the Mg(OH)_2_ product with varying concentrations of PVP added. The results indicate that when the content of surfactant PVP in the electrolyte increases, the ratio I(001)/I(101) continues to increase, while the ratio I(110)/I(001) keeps decreasing, suggesting that the addition of PVP promotes the growth of the (001) nonpolar surface. The peak intensity ratios of I(001)/I(101) and I(110)/I(001) are close at the content of 3 g·L^−1^, indicating that the growth of the (001) nonpolar surface is facilitated by the addition of PVP. The peak intensity ratios are close to each other, indicating that the growth of Mg(OH)_2_ crystals develops uniformly in all directions. As the PVP content in the electrolyte increased from 3 g·L^−1^ to 12 g·L^−1^, the ratio I(001)/I(101) keeps increasing, due to the fact that Mg(OH)_2_ preferentially grows on the (001) nonpolar face. Meanwhile, significant amounts of PVP adsorb on the (001) face, limiting the growth in the (101) face direction.

**Figure 5:**
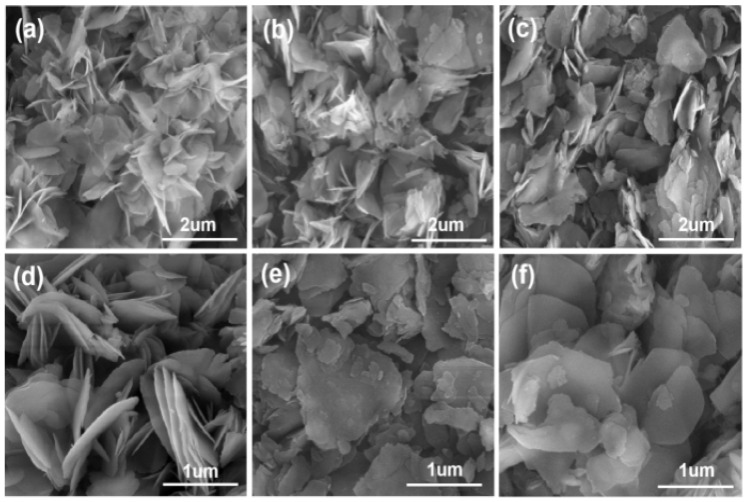
The microscopic morphology of Mg(OH)_2_ prepared by electrodeposition with surfactant in the electrolyte: (**a**) 3 g·L^−1^ PVP, (**b**) 9 g·L^−1^ PVP, (**c**) 12 g·L^−1^ PVP, (**d**) 3 g·L^−1^ PEG, (**e**) 6 g·L^−1^ PEG, and (**f**) 12 g·L^−1^ PEG.

**Figure 6:**
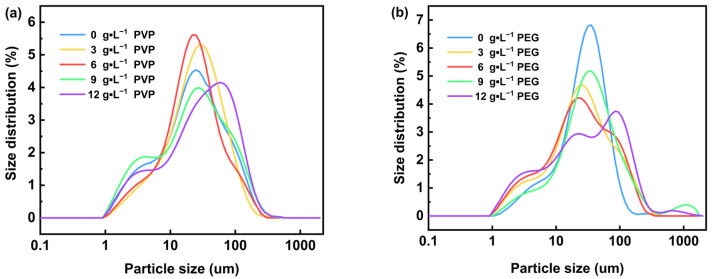
The particle size distribution of the prepared Mg(OH)_2_ with PVP (**a**) and PEG (**b**) in the electrolyte.

Figure 5 shows the microscopic morphology of Mg(OH)_2_ prepared by electrodeposition with varying concentrations of surfactant added to the electrolyte. Figure 4a–c show that with the gradual increase in PVP content in the electrolyte, from 3 g·L^−1^ to 12 g·L^−1^, the irregular flakes of Mg(OH)_2_ were gradually dispersed, and the phenomenon of agglomeration was obviously reduced. However, the irregular flake structure of Mg(OH)_2_ products remains unchanged; thus, the addition of PVP has little effect on changing the flake morphology of Mg(OH)_2_ prepared by the electrochemical method, and the primary impact is to minimize the agglomeration phenomenon between the flake particles. The particle size distribution of Mg(OH)_2_ samples prepared with PVP are shown in Figure 6. With the increasing concentration of PVP in the electrolyte, the particle size distribution range of Mg(OH)_2_ shows an overall increasing trend, becoming more uniform and concentrated at 6 g·L^−1^. Therefore, the crystal growth of Mg(OH)_2_ can be significantly influenced by the addition of PVP. Additionally, PVP effectively reduces the agglomeration phenomenon of Mg(OH)_2_ particles and optimizes its particle size distribution.

### 3.3. Effect of PEG on the Properties of Electrodeposition Product

Figure 4b demonstrates the X-ray diffraction results of the electrodeposited samples with different contents of PEG added to the electrolyte. The diffraction peaks of Mg(OH)_2_ with typical hexagonal structure can be observed; these peaks correspond to the (001), (100), (101), (102), (110), (111), (103), and (200) crystallographic planes as outlined in the standard JCPDS Card No. 07-0239, while the diffraction peaks of other phases were not detected. The 2θ values of the strongest diffraction peaks (h k l) were located at 18.52°, 37.98°, and 58.66°, the corresponding diffracted crystal planes of which were (001), (101), and (110), respectively. With the increase in PEG content in the electrolyte from 3 g·L^−1^ to 12 g·L^−1^, the intensities of the (001) crystal plane and (101) diffraction peaks of the Mg(OH)_2_ products exhibited a trend of initially increasing and then decreasing, and the crystallinity of the products was optimal at a PEG content of 6 g·L^−1^. This suggests that a moderate amount of PEG can effectively promote Mg(OH)_2_ crystallization, but an excessive amount of PEG may have an inhibitory effect on crystal growth. Figure 4d shows that the I(001)/I(101) ratio initially increases and then decreases with the increase in PEG content in the electrolyte. This indicates that the addition of PEG facilitates the growth of the (001) nonpolar surface; however, when the PEG concentration is increased to more than 6 g·L^−1^, the system favors the growth of the (110) polar surface, while the (001) surface is inhibited. At a PEG content of 12g·L^−1^, the peak intensity ratios of I(001)/I(101) and I(110)/I(001) were close to each other, which indicated that a more balanced growth of Mg(OH)_2_ crystals, with the development of crystal faces being relatively uniform.

Figure 5d–f show that with the increase in PEG content in the electrolyte from 3 g·L^−1^ to 12 g·L^−1^, the irregular lamellar grains of Mg(OH)_2_ gradually approached the near-rounded lamellar morphology. Concurrently, the agglomeration phenomenon between the grains was further improved. The best dispersion of the samples occurs at a PEG content of 12 g·L^−1^ in the electrolyte. This indicates that PEG not only improves the flaky structure of electrochemically prepared Mg(OH)_2_, but also significantly reduces the inter-granular agglomeration phenomenon within the samples. Compared with PVP, PEG showed a more significant effect in modulating the morphology of Mg(OH)_2_.

It can be seen from Figure 6b that with the increase in PEG content in the electrolyte, the half-peak width in the particle size distribution of the prepared Mg(OH)_2_ gradually increases. Specifically, the particle size increase was more obvious when the PEG content was greater than 6 g·L^−1^. This may be attributed to the fact that the addition of PEG promoted the growth of irregular flaky grains to nearly round flaky grains of Mg(OH)_2_, resulting in a gradual increase in the particle size of the samples. This result further confirms the effectiveness of PEG in regulating the crystal growth and morphology of Mg(OH)_2_.

In summary, the addition of PEG as a surfactant in the electrolyte had a significant effect on the crystal growth, microscopic morphology, and particle size distribution of Mg(OH)_2_. The appropriate amount of PEG could effectively promote the crystallization of Mg(OH)_2_ and optimize its microformat, transforming it from irregular flakes to nearly round flakes, as well as significantly reducing the inter-particle agglomeration phenomenon. In addition, the addition of PEG also led to a gradual increase in the particle size of Mg(OH)_2_, which was closely related to its role in regulating the crystal growth direction. These results suggest that PEG can be used to modulate the morphology of Mg(OH)_2_ prepared by the electrochemical method. This morphological evolution is attributed to the dual roles of PEG in selective crystal face adsorption and steric template-guided growth. The nucleation of Mg(OH)_2_ initiates from [Mg(OH)_6_]^4−^ octahedral structures, where six OH^−^ groups occupy the octahedral vertices. In the presence of PEG, the surfactant adsorbs onto specific crystallographic planes of Mg(OH)_2_ via hydrogen bonding between its hydroxyl groups and surface OH− groups [19,21]. This preferential adsorption selectively inhibits the growth of the (001) basal plane, leading to anisotropic growth along the [100] and [010] directions. Consequently, the morphology evolves from randomly oriented flakes to well-facetted hexagonal platelets, as observed in SEM analyses. Long-chain PEG molecules act as soft templates during the early stages of nucleation. PEG chains guide the directional assembly of Mg(OH)_2_ nuclei, promoting the formation of layered structures. This templating effect is evident in the gradual increase in particle size with PEG concentration, as larger aggregates form along the polymer chain axis. The steric hindrance provided by PEG also prevents excessive agglomeration, resulting in improved particle dispersion. These findings highlight the potential of PEG as a versatile morphology regulator in electrochemical synthesis, offering precise control over Mg(OH)_2_ properties for diverse applications. Hexagonal platelets exhibit enhanced flame-retardant efficiency due to their high aspect ratio and uniform dispersion in polymer matrices [4,22]. The reduced agglomeration and increased surface area of PEG-modulated Mg(OH)_2_ improve its performance in wastewater treatment [7,18].

## 4. Conclusions

This paper investigates the effects of PVP and PEG as additives to the process parameters and product properties of Mg(OH)_2_ prepared by electrodeposition with MgCl_2_. The results indicate that incorporating surfactants into the electrolyte significantly influences cell voltage, pH in the cathode chamber, current efficiency, and energy consumption during magnesium hydroxide preparation. When the addition amount of surfactant PVP is 6 g·L^−1^, an optimal current efficiency of 93.86% and the lowest energy consumption value of 4.15 kWh t^−1^ are achieved. Similarly, the addition of PEG follows the same trend. Under electrodeposition conditions, the incorporation of PVP enhances the dispersibility of magnesium hydroxide, resulting in an increase in particle size. Additionally, the addition of an appropriate amount of PEG facilitates the transformation of Mg(OH)_2_ from an irregular morphology to a nearly circular sheet-like structure. This study presents a novel electrochemical technology for preparing Mg(OH)_2_ using magnesium chloride salt and provides a theoretical foundation for better control over product morphology.

## Figures and Tables

**Table 1:** The components of bischofite.

Composition	MgCl_2_·6H_2_O	Insoluble Matter	SO_4_^2−^	H_2_O	Na^+^	K^+^	Li^+^	Ca^2+^
Content %	96.85	0.06	0.20	1.88	0.12	0.09	0.02	0.05

**Table 2:** The specific information of the diaphragm.

Model	Material	Thickness mm ± 10%	Weight g/m^2^ ± 5%	PH Range	Temperature °C	Air Permeability L/m^2^·s
303C	pp	1.75	1070	0–14	≤90	35 ± 7

## Data Availability

The original contributions presented in this study are included in the article. Further inquiries can be directed to the corresponding authors.
